# Molecular biomarkers of Alzheimer's disease: progress and prospects

**DOI:** 10.1242/dmm.031781

**Published:** 2018-05-08

**Authors:** Tammaryn Lashley, Jonathan M. Schott, Philip Weston, Christina E. Murray, Henny Wellington, Ashvini Keshavan, Sandrine C. Foti, Martha Foiani, Jamie Toombs, Jonathan D. Rohrer, Amanda Heslegrave, Henrik Zetterberg

**Affiliations:** 1 Queen Square Brain Bank for Neurological Disorders, Department of Molecular Neuroscience, UCL Institute of Neurology, London WC1N 3BG, UK; 2 Dementia Research Centre, UCL Institute of Neurology, London WC1N 3BG, UK; 3 Department of Molecular Neuroscience, UCL Institute of Neurology, Queen Square, London WC1N 3BG, UK; 4 UK Dementia Research Institute, London WC1N 3BG, UK; 5 Institute of Neuroscience and Physiology, Department of Psychiatry and Neurochemistry, the Sahlgrenska Academy at the University of Gothenburg, Mölndal S-431 80, Sweden; 6 Clinical Neurochemistry Laboratory, Sahlgrenska University Hospital, Mölndal S-431 80, Sweden

**Keywords:** Alzheimer's disease, Biomarkers, Cerebrospinal fluid, Blood, Plasma, Serum, Tau, Amyloid, Neurofilament, Neurogranin

## Abstract

The neurodegenerative disorder Alzheimer's disease is characterised by the formation of β-amyloid plaques and neurofibrillary tangles in the brain parenchyma, which cause synapse and neuronal loss. This leads to clinical symptoms, such as progressive memory deficits. Clinically, these pathological changes can be detected in the cerebrospinal fluid and with brain imaging, although reliable blood tests for plaque and tangle pathologies remain to be developed. Plaques and tangles often co-exist with other brain pathologies, including aggregates of transactive response DNA-binding protein 43 and Lewy bodies, but the extent to which these contribute to the severity of Alzheimer's disease is currently unknown. In this ‘At a glance’ article and poster, we summarise the molecular biomarkers that are being developed to detect Alzheimer's disease and its related pathologies. We also highlight the biomarkers that are currently in clinical use and include a critical appraisal of the challenges associated with applying these biomarkers for diagnostic and prognostic purposes of Alzheimer's disease and related neurodegenerative disorders, also in their prodromal clinical phases.

## Abstract (precis)

**Summary:** This ‘At a glance’ article summarises the molecular biomarkers of Alzheimer's and related diseases, highlighting the challenges and opportunities for diagnostic and prognostic applications.

## Introduction

Neurodegenerative dementias constitute a broad category of brain diseases that are characterised by a typically gradual decline in cognitive function, ultimately leading to increased mortality. The most common type of dementia is Alzheimer's disease (AD), which accounts for 50-70% of prevalent neurodegenerative dementia cases (Winblad et al., 2016). AD causes a progressive loss of cognitive abilities with short-term memory impairment being the most typical initial symptom. However, there are also atypical clinical presentations of AD, such as logopenic variant primary progressive aphasia (see Glossary, Box 1) or posterior cortical atrophy (Mattsson et al., 2016a). Alongside AD, there are many other dementia-causing neurodegenerative diseases that might be important for differential diagnoses (Schott and Warren, 2012).

**Figure:**
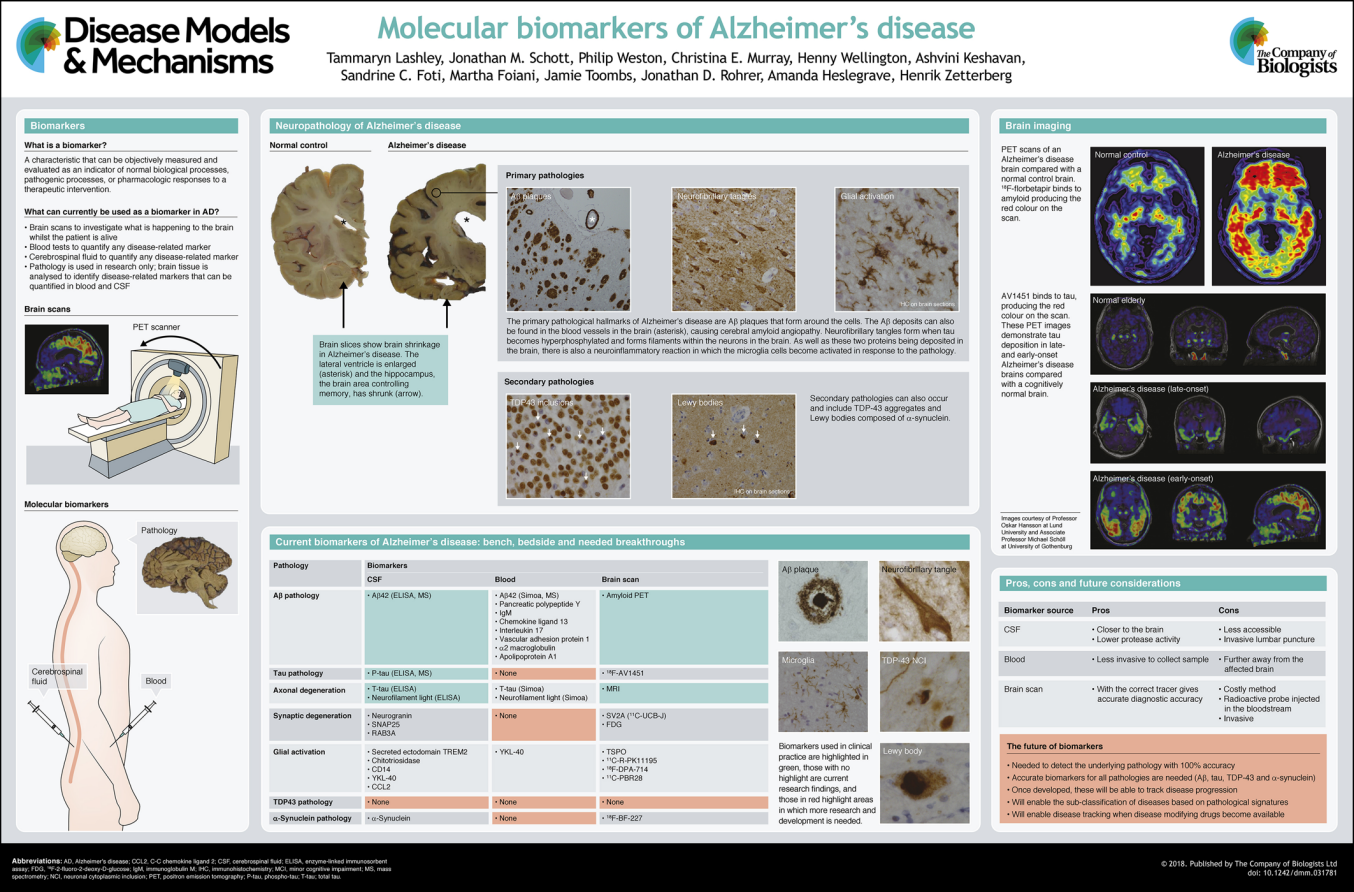

Traditionally, a dementia diagnosis is based on the history of the illness, the pattern of cognitive deficits, and on additional parameters assessed through clinical investigations, including blood tests and structural imaging of the brain, to rule out nondegenerative causes of the symptoms. Increasingly, and with the prospect of disease modification, there has been a shift towards the use of biomarkers (Dubois et al., 2014) to diagnose specific forms of dementia earlier, also in the pre-dementia stages of the disease, and with more specificity. Currently, a definite diagnosis of a neurodegenerative dementia requires histopathological confirmation at autopsy, as different degenerative dementia-causing brain disorders are characterised by more or less distinct pathologies (Hyman et al., 2012). A striking common feature of most neurodegenerative dementias is the presence of aggregates or inclusions of misfolded endogenous proteins in the brain extracellular matrix or within neurons and other brain cell types (Kovacs, 2016). This common feature classifies these dementias as proteopathies (Box 1) (Walker and Jucker, 2015).

**Box 1. Glossary**

**Amyloid positron emission tomography:** an imaging technique that visualises amyloid senile plaques in the living human brain.

**Axonal degeneration:** a progressive degeneration and loss of axons.

**β-amyloid:** the cleavage product of amyloid precursor protein and the main constituent of senile (amyloid) plaques in Alzheimer's disease.

**Cerebral amyloid angiopathy:** a form of blood vessel disorder in which amyloid deposits form in the walls of the blood vessels of the central nervous system, affecting blood flow.

**Cerebral β-amyloidosis:** β-amyloid plaque pathology in the brain.

**Creutzfeldt-Jakob disease (CJD):** a rapidly progressive neurodegenerative disease caused by self-propagating aggregation of a normal brain protein (prion protein).

**Dementia with Lewy bodies (DLB):** a progressive brain disorder in which Lewy bodies, deposits of the protein α-synuclein build up in areas of the brain that regulate behaviour, cognition and movement.

**Diffuse leukoaraiosis:** an abnormal change in appearance of white matter near the lateral ventricles of the brain.

**Frontotemporal dementia (FTD):** the clinical presentation of frontotemporal lobar degeneration, which is characterised by progressive neuronal loss predominantly involving the frontal or temporal lobes.

**Lacunar infarction:** the most common type of stroke that results from the occlusion of small penetrating arteries that provide blood to the brain's deep structures.

**Logopenic variant primary progressive aphasia:** a language disorder that involves changes in the ability to speak, read, write and understand what others are saying.

**Parkinson's disease dementia:** Parkinson's disease that later progresses into dementia.

**Progressive supranuclear palsy:** a tauopathy that often starts with Parkinson-like symptoms but then progresses to involve other brain regions.

**Proteopathies:** a class of diseases in which certain proteins become structurally abnormal, and thereby disrupt the function of cells, tissues and organs of the body.

**Senile plaques:** extracellular deposits of β-amyloid.

**Superficial central nervous system siderosis:** a disease of the brain resulting from chronic iron deposition in neuronal tissues associated with cerebrospinal fluid.

**Synaptic degeneration:** a progressive degeneration and loss of synapses.

**Synucleinopathies:** diseases in which α-synuclein inclusions accumulate inside neurons and other cell types of the brain.

**Tau:** a structural protein in axons that may form neurofibrillary tangles if truncated and hyperphosphorylated.

**Tauopathies:** neurodegenerative diseases in which abnormal inclusions of tau accumulate inside neurons.

**Temporo-parietal association cortices:** the cerebral cortex outside the primary areas in the temporal and parietal lobes.

**Vascular dementia (VaD):** a form of dementia caused by cerebrovascular disease, resulting in neuronal injury and degeneration.

Neuropathologically, AD is characterised by: (1) neuronal loss in specific brain regions – notably the medial temporal lobe structures and the temporo-parietal association cortices (Box 1); (2) intraneuronal neurofibrillary tangles composed of aggregated and often truncated and hyperphosphorylated tau protein (Box 1); and (3) extracellular neuritic plaques, which consist of deposits of β-amyloid (Box 1) peptides, mainly its 42-amino-acid isoform (Blennow et al., 2006) (see poster). There are other neurodegenerative diseases with symptoms that might overlap with AD, such as frontotemporal dementia (FTD; Box 1), in which inclusions can consist of several different proteins, most typically tau (MAPT) and/or transactive response DNA-binding protein 43 (TDP-43; TARDBP); Parkinson's disease dementia (PDD; Box 1) and dementia with Lewy bodies (DLB; Box 1), in which α-synuclein inclusions represent an important part of the pathology (Box 1). These neurodegenerative pathologies often present with a considerable degree of co-morbidity, with several different pathological changes co-occurring in the same brain tissue, indicating that pathologically deposited proteins might interact and be influenced by other factors to cause cognitive decline and other clinical symptoms (Lashley et al., 2008). For example, cerebral small vessel disease, which might be caused by several different pathological processes, including lacunar infarction, diffuse leukoaraiosis and cerebral amyloid angiopathy (Box 1), can both cause dementia and co-exist with degenerative dementias, thus influencing their severity and phenotype. Because clinical phenotypes of dementia can be caused by different pathological changes, sometimes interacting with each other, it is important to develop biomarkers that can diagnose these changes to improve the possibility to monitor and treat the underlying cause.

In this ‘At a glance’ poster, we show the different neuropathological changes that might underlie neurodegenerative dementias, especially AD, and discuss the currently available molecular fluid- and imaging-based biomarkers for each pathology. We also discuss why biomarkers are necessary in the clinic, the challenges encountered when using them clinically, which patient populations the different biomarkers could serve, and the issues relating to the implementation of standardised sampling and handling protocols. The biomarker concept with special focus on neurodegenerative dementias is detailed in the far-left panel of the poster. The discussion of structural or functional imaging biomarkers is beyond the scope of this article, but we refer the interested reader to an excellent recent review on these biomarkers (Rathore et al., 2017).

## Biomarkers for β-amyloid pathology

The 42-amino-acid isoform of β-amyloid (Aβ42) is a major component of senile plaques (Box 1) and contributes to cerebral amyloid angiopathy in AD (Masters et al., 1985) (see poster). Aβ42 is a cleavage product of type I transmembrane amyloid precursor protein (APP) with no known physiological function. It is released from neurons when APP is cleaved by β- and γ-secretases in synaptic vesicles. APP is metabolised by many cell types, but Aβ42 secretion is by far the highest from neurons; its neuronal secretion also appears to depend on synaptic activity (Cirrito et al., 2005).

### Cerebrospinal fluid biomarkers

Aβ42 concentration can be measured in the cerebrospinal fluid (CSF) by antibody-based techniques, such as enzyme-linked immunosorbent assay (ELISA), as well as by antibody-independent techniques, such as mass spectrometry (Kuhlmann et al., 2016). AD patients have decreased concentrations of Aβ42 in their CSF, a finding that has been well verified and replicated by many studies (Olsson et al., 2016). This decrease reflects the sequestration of Aβ42 in senile plaques in the brain, as evidenced by autopsy and by *in vivo* amyloid positron emission tomography (PET) imaging (Box 1) studies in patients (Blennow et al., 2015). Reduced levels of Aβ42 can be detected in the CSF of patients with mild cognitive impairment (MCI), as well as in the pre-clinical stages of AD (Bateman et al., 2012; Olsson et al., 2016). A plaque pathology-associated decrease in Aβ42 concentration in the CSF is also frequently seen in DLB, another neurodegenerative dementia that is very commonly accompanied by cerebral Aβ aggregation (Abdelnour et al., 2016).

### Blood biomarkers

It has been difficult to establish robust blood biomarkers for Aβ pathology. Aβ proteins can be measured in plasma, but their correlation with cerebral β-amyloidosis (Box 1) is absent or weak when the latter is assessed immunochemically (Olsson et al., 2016). Plasma Aβ concentrations are probably influenced by its secretion from platelets and from other extracerebral tissues (Zetterberg, 2015). Nevertheless, three recent studies have reported a clinically significant correlation between plasma Aβ concentrations and cerebral β-amyloidosis measured by mass spectrometry (Kaneko et al., 2014; Nakamura et al., 2018; Ovod et al., 2017). A similar result has been obtained using an ultrasensitive assay (Janelidze et al., 2016). The results of these papers are promising and warrant further validation. Other studies have reported that various plasma proteins (including pancreatic polypeptide Y, immunoglobulin M, chemokine ligand 13, interleukin 17, vascular cell adhesion protein 1, α2-macroglobulin, apolipoprotein A1 and complement proteins) are associated with Aβ42 burden in the brain, irrespective of the clinical stage of AD (Burnham et al., 2016; Westwood et al., 2016; Voyle et al., 2015). However, these data should be interpreted with caution, as they are derived from multimarker panels and have not been replicated or examined in relation to other neurodegenerative dementias. Further, we are currently lacking a mechanistic understanding of these associations.

### Biomarkers for PET

The first chemical probe for amyloid PET was an ^11^C-labelled modified derivative of the amyloid-binding histological dye thioflavin-T called Pittsburgh Compound-B (PiB, also known as ^11^C-PiB). This probe is retained in cortical brain regions of AD patients compared with healthy controls, with retention in the cerebellum used as a reference region (Klunk et al., 2004). The increased retention of PiB in cortical brain regions of AD patients has since been verified in many scientific reports (reviewed in Blennow et al., 2015). However, the short half-life of ^11^C hinders the use of ^11^C-PiB outside of expert research centres that have access to an on-site cyclotron to generate the probe, as well as to radiochemistry expertise. As a result, ^18^F-labelled probes have been developed that have a half-life of ∼110 min. This longer half-life enables the centralised production of this probe and its regional distribution to medical centres that have a PET scanner. Three ^18^F-PET amyloid tracers are licensed for clinical use: ^18^F-florbetapir (Amyvid), ^18^F-flutemetamol (Vizamyl) and ^18^F-florbetaben (Neuraceq), all of which have shown good correlation with amyloid plaque burden at autopsy (Morbelli and Bauckneht, 2018), although they generally show higher levels of nonspecific binding to white matter compared with ^11^C-PiB.

## Biomarkers for tau pathology

Abnormally phosphorylated and truncated tau proteins are the major component of neurofibrillary tangles in AD and other tauopathies (Box 1) (Grundke-Iqbal et al., 1986) (see poster). The normal function of tau is to bind to and stabilise microtubules in neuronal axons (Zetterberg, 2017), a process that is inhibited when tau becomes phosphorylated.

### CSF biomarkers

Tangle-containing neurons release phosphorylated tau, which can be measured in the CSF by ELISA, using antibody combinations that specifically recognise mid-domain phospho-tau (P-tau) epitopes. AD patients have increased concentrations of P-tau in their CSF (Olsson et al., 2016). However, P-tau concentration in the CSF correlates weakly with neurofibrillary tangle pathology in the brain of AD patients (Buerger et al., 2006; Seppala et al., 2012). This finding was replicated in a recent tau PET imaging study of AD patients (Chhatwal et al., 2016), although the results are less clear than the association of CSF Aβ42 with amyloid PET.

A major outstanding research question is why other tauopathies, including some forms of FTD and associated disorders like progressive supranuclear palsy (Box 1), do not show increased P-tau concentration in the CSF, at least not as robustly as in AD (Zetterberg, 2017). It is possible that disease-specific phosphorylation of tau occurs in these disorders, or that tau is processed or truncated in a way that is not recognised by the available assays. Another potential explanation for why increased CSF P-tau is specific to AD is that this particular pathological change is simply more extensive and severe in AD than it is in other tauopathies. CSF P-tau is currently considered to be the most specific biomarker for AD. Except for herpes encephalitis and superficial CNS siderosis (Box 1), no other condition features a systematic increase in this biomarker (Zetterberg, 2017).

### Blood biomarkers

To date, no reliable blood biomarkers for neurofibrillary tangle pathology have been identified. However, recent studies have reported increased P-tau concentrations in blood-borne neuron-derived exosomes (Shi et al., 2016; Winston et al., 2016). In this assay, the exosomes are isolated from serum using antibodies directed against neuron-enriched proteins. The isolated exosomes are then washed and lysed and their tau content is measured using immunochemical assays. Although new, this technique represents a promising approach for P-tau measurements in blood.

### Biomarkers for PET

PET tracers (also known as probes) have been developed to visualise tau inclusions *in vivo* in patients. One of these tracers, ^18^F-AV1451, binds to tau aggregates in AD (Marquié et al., 2015), can differentiate AD patients from healthy controls (Schöll et al., 2016) and correlates with regional changes in brain metabolism in different clinical variants of AD (Ossenkoppele et al., 2016) (see poster). However, AV1451 does not reliably bind to all pathological isoforms of tau, nor does its binding reliably correlate with pathological tau load (Sander et al., 2016). Similar results have been published for other tau PET probes, although they might recognise different forms of tau deposits because of differences in their structure and binding properties (Kolb and Andres, 2017). Preliminary evidence indicates that tau measurements in the CSF and via PET correlate in the dementia stage of AD, but less clearly in the preclinical and mild cognitive impairment stages of the disease (Mattsson et al., 2017). However, this correlation is not as well established as that between CSF and PET biomarkers for Aβ pathology.

## Biomarkers for axonal degeneration

Axonal degeneration (Box 1) is a key feature of AD, and is more closely linked to the onset of cognitive decline than Aβ pathology is (see poster, ‘Biomarkers’). In fact, according to some models, the onset of neurodegeneration marks the beginning of the toxic phase of Aβ pathology in the pathogenesis of AD (Jack and Holtzman, 2013).

### CSF biomarkers

Total tau (T-tau), measured using antibodies against mid-domain tau epitopes that are not phosphorylated, can be used as a general marker of axonal degeneration or injury in AD. AD patients have increased concentrations of T-tau in their CSF (Olsson et al., 2016), and the higher the increase, the more intense the neurodegenerative process (Wallin et al., 2010). However, increased levels of CSF T-tau are not specific to AD; the increase is also seen, for example, in Creutzfeldt-Jakob disease (CJD; Box 1) (Riemenschneider et al., 2003) and following stroke (Hesse et al., 2001). Assays have also been developed to quantify visinin-like protein 1 (VLP-1; VSNL1) and members of the fatty acid-binding protein (FABP) family in the CSF. VLP-1 and FABP proteins are enriched in neurons, but their association with AD is less strong than that of CSF T-tau (Olsson et al., 2016). Neuron-specific enolase (NSE; ENO2) has also been proposed as a candidate biomarker for neuronal loss in AD, but its association with AD is weak and clinically irrelevant (Olsson et al., 2016). In addition, the results of NSE tests are easily confounded by blood contamination, because NSE (despite its name) is highly expressed in erythrocytes (Ramont et al., 2005).

Another CSF biomarker for axonal degeneration is neurofilament light (NF-L; NEFL), which is a structural protein present in long axons (Zetterberg, 2016). The concentration of NF-L is increased in the CSF of AD patients, especially so in those with rapid disease progression (Zetterberg et al., 2016). However, increased NF-L in the CSF is not specific to AD, and is detected in other dementias, with the highest concentrations seen in FTD and in vascular dementia (VaD; Box 1) (de Jong et al., 2007; Landqvist Waldö et al., 2013; Sjogren et al., 2000). These results were recently confirmed in a large retrospective analysis of data from the Swedish Dementia Registry (Skillback et al., 2014), as well as in atypical parkinsonian disorders (Hall et al., 2012; Magdalinou et al., 2015). The highest CSF concentrations of NF-L are seen in CJD, as is the case for T-tau (Steinacker et al., 2016; van Eijk et al., 2010).

### Blood biomarkers

CSF assays for T-tau and NF-L have recently been redeveloped into ultrasensitive blood tests using single molecule array (Simoa) technology (Andreasson et al., 2016). Serum and plasma NF-L concentrations correlate with their concentrations in the CSF (correlation coefficients of 0.75 to 0.97), and most measurements in the CSF (increased NF-L concentrations in AD, FTD, VaD and in atypical parkinsonian disorders) have been replicated in blood (Zetterberg, 2016). For T-tau, such correlation is less clear, but promising. First, for unknown reasons, tau concentrations are higher in plasma than in serum (H.Z., unpublished). Second, the correlation with the corresponding CSF concentration is either absent (Zetterberg et al., 2013) or weak (Mattsson et al., 2016b). In AD, plasma T-tau levels are increased, but less so than in the CSF, and there is no detectable increase in the MCI stage of the disease (Mattsson et al., 2016b; Zetterberg et al., 2013).

### PET biomarkers

There are presently no PET probes for axonal degeneration. There will likely not be any in the near future, as there are no targetable molecular assemblies that are specific to axonal degeneration and which could function as anchors for PET probe binding.

## Fluid biomarkers for synaptic degeneration

In its earliest clinical phase, AD characteristically and consistently causes memory impairment. Mounting evidence suggests that memory impairment begins with subtle alterations to synaptic efficacy in the hippocampus, prior to frank neuronal degeneration (see poster). A reduction in synapse number is associated with numerous brain disorders, and with AD in particular (Selkoe, 2002).

### CSF biomarkers

Neurogranin (Ng; NRGN) is a dendritic protein enriched in neurons that is involved in long-term potentiation of synapses, particularly so in the hippocampus and the basal forebrain (Represa et al., 1990). Recently, several independent studies have shown that the CSF concentration of Ng is increased in AD (Hellwig et al., 2015; Kester et al., 2015; Kvartsberg et al., 2015a,b; Thorsell et al., 2010), but not in other neurodegenerative disorders (Wellington et al., 2016). Moreover, studies showed a quantitative correlation between the magnitude of Ng increase and the severity of cognitive decline, brain atrophy and reduction in glucose metabolism in the prodromal stage of the disease (Portelius et al., 2015; Tarawneh et al., 2016). Currently, CSF Ng is the best-established CSF biomarker for synapse loss or dysfunction associated with AD, although other promising markers of this pathological change are being characterised in single-centre studies awaiting independent replication. These markers include synaptosomal-associated protein 25 (SNAP25) and Ras-related protein RAB3A (Bereczki et al., 2016; Brinkmalm et al., 2014).

### Blood biomarkers

There are so far no reliable blood biomarkers for synaptic pathology. Plasma Ng has been explored as a candidate marker in this context, but its concentration remained unchanged in AD patients compared with cognitively healthy controls (De Vos et al., 2015). Most likely, the extracerebral expression of Ng constitutes the major source of Ng in plasma (De Vos et al., 2015), confounding potential differences between the healthy and AD groups.

### PET biomarkers

In a recent report, the synaptic vesicle glycoprotein 2A (SV2A) radioligand ^11^C-UCB-J combined with PET was used to quantify synaptic density in the living human brain (Finnema et al., 2016). The probe had excellent imaging properties and was sensitive enough to detect synaptic loss in patients with temporal lobe epilepsy. Its utility in AD, however, remains to be established. Another technique to monitor synaptic loss in neurodegenerative dementias is ^18^F-2-fluoro-2-deoxy-D-glucose (FDG)-PET. This method detects brain region-specific impairment of cerebral glucose metabolism in neurodegenerative diseases (Payoux and Salabert, 2017). A recent systematic review and meta-analysis revealed varying diagnostic accuracies of FDG-PET, with sensitivities between 46% and 95%, while the specificities were between 29% and 100%, and the conclusion was that the method in its current stage of development cannot be recommended for clinical use in AD, with more research recommended (Smailagic et al., 2015).

## Biomarkers for glial activation

Glial cells in the brain are astrocytes, the star-shaped cells that provide neurons with nutrients, form part of the blood-brain barrier and take part in repair mechanisms following CNS injury, and microglia, the resident macrophages of the brain, constituting the main form of active immune defence in the CNS. Most often, both cell types are activated in parallel, and glial activation has been linked to deficits in neuronal function and to synaptic plasticity in AD. The recent discovery of a genetic link between AD and variants of the triggering receptor expressed on myeloid cells 2 (*TREM2*; *TREML2*) gene (Guerreiro et al., 2013; Jonsson et al., 2013), which is selectively expressed on microglia in the CNS (Lue et al., 2015; Takahashi et al., 2005), has reignited the interest in identifying biomarkers of glial activation (see poster).

### Cerebrospinal fluid biomarkers

Recent reports suggest that the concentrations of the secreted ectodomain of TREM2 are increased in the CSF of AD patients. This increase is disease specific and correlates with elevated CSF levels of T-tau and P-tau (Heslegrave et al., 2016; Piccio et al., 2016; Suárez-Calvet et al., 2016). These findings are supported by numerous studies that report the increased CSF concentrations of several other astrocyte-, microglia- and/or macrophage-derived proteins, including chitotriosidase (Mattsson et al., 2011; Watabe-Rudolph et al., 2012), CD14 (Yin et al., 2009) and YKL-40 (CHI3LI) (Craig-Schapiro et al., 2010; Olsson et al., 2013). Another glial marker, the C-C chemokine receptor 2, is expressed on monocytes, and one of its ligands, C-C chemokine ligand 2 (CCL2), which can be produced by microglia, is also present in increased concentrations in the CSF of AD patients (Corrêa et al., 2011; Galimberti et al., 2006a,b). Most studies suggest that these increases in glial proteins in AD are modest, with concentration ranges overlapping extensively between cases and controls, particularly when compared with the more prominent changes seen in ‘traditional’ neuroinflammatory conditions, such as multiple sclerosis (Öhrfelt et al., 2016) or human immunodeficiency virus (HIV)-associated neurocognitive dysfunction (Peluso et al., 2017). It should also be noted that all of the above-mentioned proteins, except TREM2, can also be released from activated astrocytes. Thus, microglial and astrocytic activation in AD are difficult to tease apart using only CSF-based biomarkers. This problem might not have any practical implications, as microglial and astrocytic activation are tightly linked and, as markers of the two processes, could potentially be used interchangeably to track AD.

### Blood biomarkers

When biomarkers of microglial activation, such as those mentioned above, are measured in plasma or serum, their concentrations are similar to those in the CSF and not 100-fold lower as would have been expected if they were CNS derived (Craig-Schapiro et al., 2010). This probably reflects their release from monocytes and macrophages in peripheral blood rather than reflecting CNS-related changes. However, a few studies indicate that YKL-40 concentration is slightly increased in the plasma of AD patients (Olsson et al., 2016); the overlap between AD patients and cognitively normal controls was, however, too large for the marker to be used clinically.

### PET biomarkers

The mitochondrial translocator protein (TSPO) is known to be upregulated in activated microglia. Accurate visualisation and quantification of microglial density by PET imaging using the TSPO probe [^11^C]-R-PK11195 has been challenging owing to the limitations of the probe, mainly its low brain permeability and the abundant expression of TSPO in extracerebral tissues. A number of new TSPO probes (e.g. [^18^F]-DPA-714 and [^11^C]-PBR28) have been evaluated in rodent and nonhuman primate models, but the literature on their clinical usefulness remains scant (Lagarde et al., 2017).

## Biomarkers for TDP-43 pathology

Hyperphosphorylated TDP-43 proteinopathy occurs in ∼50% of FTD patients and has recently been described both in studies of ageing and in association with the cognitive impairment of ageing patients, especially in the context of tau and Aβ pathology (James et al., 2016), providing some overlap with AD (see poster).

### CSF biomarkers

Total TDP-43 can be measured in the CSF but, unfortunately, most of the protein appears to be blood derived, as it is ubiquitously expressed throughout the body. Thus, its concentration in the CSF does not reflect the presence of TDP-43 proteinopathy in the brain. Moreover, its levels in the CSF of FTD patients are also unaltered (Feneberg et al., 2014), further disqualifying this protein as a potential CSF biomarker.

### Blood biomarkers

No reliable blood test for TDP-43 pathology in the CNS exists. Given the ubiquitous expression of TDP-43 throughout the body, brain inclusion-specific pathologic forms of TDP-43 would have to be targeted in the blood for this to become a feasible biomarker project.

### Biomarkers for PET

There are presently no PET probes for TDP-43 inclusions. In 2016, the Amyotrophic Lateral Sclerosis Association announced a Grand Challenge grant to develop TDP-43 inclusion probes but, so far, there are no published data on this topic.

## Biomarkers for α-synuclein pathology

The presynaptic neuronal protein α-synuclein can misfold and form seeds that can aggregate further into inclusions that are called Lewy bodies. These inclusions are characteristic of Parkinson's disease (PD) and of DLB (Mollenhauer et al., 2010), but also often feature in AD (Schneider et al., 2009) (see poster).

### Cerebrospinal fluid biomarkers

In PD and in other synucleinopathies (Box 1), α-synuclein concentrations in the CSF are typically lower than in healthy controls (Hall et al., 2012; Mollenhauer et al., 2011), whilst in AD and CJD, its concentrations are increased and correlate with T-tau, indicating that α-synuclein might also be a nonspecific marker of neurodegeneration (Mollenhauer et al., 2011; Öhrfelt et al., 2009; Slaets et al., 2014; Tateno et al., 2012; Wennström et al., 2013). Increased CSF levels of α-synuclein have also been reported in DLB, where a competition might exist between the aggregation of α-synuclein into Lewy bodies and its release from the degenerating synapses, which complicates data interpretation (Kapaki et al., 2013). In agreement with this hypothesis, a recently published multiple reaction monitoring mass spectrometry assay revealed significantly increased CSF concentrations of α-, β- and γ-synuclein in AD and CJD, but not in the ‘classical’ synucleinopathies like PD (Oeckl et al., 2016). Currently available assays for α-synuclein measure the total amounts of the protein and not the Lewy body-specific isoforms. The availability of sensitive and specific assays for these pathogenic isoforms would resolve the issue of having the biomarker results influenced or confounded by the release of native α-synuclein from degenerating synapses. However, there are some preliminary reports of increased concentrations of α-synuclein oligomers in the CSF of PD patients (Hansson et al., 2014; Tokuda et al., 2010). Recent studies described sensitive assays that detect the amplified biochemical signal of α-synuclein seeds that might be Lewy body derived in the CSF from PD patients but not in that from healthy controls (Fairfoul et al., 2016; Shahnawaz et al., 2016), opening a promising avenue for using CSF α-synuclein as a biomarker.

### Blood biomarkers

Because α-synuclein is highly expressed in red blood cells, blood contamination during CSF collection might limit its diagnostic value (Barbour et al., 2008; Hong et al., 2010). For the very same reason, blood tests for α-synuclein pathology in the brain might lack the specificity needed for it to be an informative clinical biomarker. Nevertheless, as peripheral Lewy body pathology, such as in the salivary gland and the gut, has been reported in PD (Uchihara and Giasson, 2016), blood or salivary tests for α-synuclein seeds might be worth exploring in the future as a biomarker of PD and other dementias associated with Lewy bodies, such as AD.

### PET biomarkers

Efforts to develop PET probes for α-synuclein inclusions are ongoing but are still in their infancy. One of the compounds currently investigated for imaging α-synuclein inclusions is the ^18^F-labelled compound BF-227 that was reported to bind to both synthetic α-synuclein aggregates as well as β-amyloid fibrils *in vitro* (Fodero-Tavoletti et al., 2009). A histopathological study demonstrated that BF-227 could stain α-synuclein-containing glial cytoplasmic inclusions in postmortem tissue (Fodero-Tavoletti et al., 2009). Moreover, a PET study with ^11^C-labelled BF-227 showed its ability to detect α-synuclein deposits in the living brains of patients with multiple system atrophy (Kikuchi et al., 2010). However, the high affinity of this radiotracer for Aβ plaques limits its usefulness for differential diagnosis (Kikuchi et al., 2010).

## Pros and cons of the different biomarker modalities

A biomarker can be defined as ‘a characteristic that is objectively measured and evaluated as an indicator of normal biological processes, pathogenic processes or pharmacologic responses to a therapeutic intervention’ (Strimbu and Tavel, 2010). Biomarkers for AD should, thus, reflect the core pathogenic findings in the brain, i.e. plaque and tangle pathology, as well as the associated pathophysiological mechanisms, i.e. axonal and synaptic degeneration (Box 1) and frequent co-pathologies, including TDP-43 and α-synuclein pathologies (see poster, ‘Brain imaging’). We have discussed three broad categories of biomarker modalities in this ‘At a glance’ paper: CSF, blood and PET, and they differ in terms of accessibility and how closely they reflect the changes in the brain (see poster). In regards to the fluids, the CSF is closer to the brain and has a lower intrinsic protease activity than blood. However, the CSF is less accessible, as sampling requires a lumbar puncture. Regarding PET, this is a much more costly method. Further, it involves injecting a radioactive probe into the blood. This probe will cross the blood-brain barrier and remain bound to its target pathologies for an unknown time period. This technique can thus also be regarded as invasive. In regards to plaque pathology, the diagnostic accuracies of the CSF and PET tests are comparable (Blennow et al., 2015). However, for the other pathologies, prospective studies that directly compare the different biomarker modalities are needed to determine whether any one marker is better at reflecting the extent of the pathology than another. Regarding blood tests, one biomarker stands out as being particularly promising: NF-L, for which robust CSF and plasma/serum correlations have been established. For this particular protein, virtually the same information can be gathered from a CSF test and a blood test (Zetterberg, 2016). For the other blood tests, more research is needed before any of these could replace the corresponding CSF or PET test (see poster). For biomarkers, fluid- and imaging-based alike, to be implemented in clinical practice, the measurement techniques have to be well standardised and give stable results over time. For CSF Aβ42 measurements, there are now certified reference methods and a reference material is under production (Kuhlmann et al., 2016). Similar work is ongoing for the CSF-based tau biomarkers. Pre-analytical standard operating procedures for CSF sampling and storage have been published (Blennow et al., 2010), and guidelines on how to interpret the results in different clinical stages of the disease are being developed (Herukka et al., 2017; Simonsen et al., 2017). Similarly, reliable quantitative analysis of amyloid PET scans acquired at multiple sites and over time requires rigorous standardisation of the acquisition protocols, subject management, tracer administration, image quality control, and image processing and analysis methods. Approaches to address these issues have been published (Schmidt et al., 2015). For most of the other candidate biomarkers discussed in this paper, such work is pending and will be required prior to the transition of these biomarkers into routine clinical practice.

## Conclusions

Disease biomarkers have been developed into clinically available methods to detect tangle and plaque pathology in the CSF and brains of AD patients, and there are also promising biomarkers to detect synaptic loss and dysfunction. Tau and Aβ biomarkers can help to diagnose AD pathology in both the prodromal and the dementia stages of the disease. Moreover, a number of additional biomarkers have been identified that detect pathological changes common to AD and other neurodegenerative proteopathies, although reliable and accurate biomarkers for TDP-43 and Lewy body pathology remain to be identified. If identified in the future, such biomarkers could be employed in longitudinal studies to track the temporal development of different pathologies during neurodegenerative disease progression, and to assess how their interactions lead to clinical symptoms. As multimorbidity appears to be common not only in AD but also in other neurodegenerative dementias, one potential future scenario is that these biomarkers could be used to subclassify the clinical syndromes in individual patients according to their pathological signature, allowing for personalised treatment.
